# Medication-overuse headache—a review of different treatment strategies

**DOI:** 10.3389/fpain.2023.1103497

**Published:** 2023-10-10

**Authors:** Abouch Krymchantowski, Carla Jevoux, Ana Gabriela Krymchantowski, Luiza Barbosa Ramos, Jackeline S. S. Barbosa, Raimundo Pereira Silva-Neto

**Affiliations:** ^1^ Headache Center of Rio, Rio de Janeiro, Brazil; ^2^ Federal University of the Parnaíba Delta, Parnaíba, Brazil

**Keywords:** medication-overuse headache, secondary headache, treatment, withdrawal, preventive medications, chronic migraine

## Abstract

Medication-overuse headache (MOH) can develop from primary headaches. MOH is usually the result of overuse of symptomatic medications. It is a noteworthy personal and societal burden. The identification and treatment of patients at risk for MOH is an essential component of MOH management. Medication overuse can be modifiable and can advance from episodic to chronic migraine. Treatment for MOH is complex, and experts in the field have varied views on the most appropriate strategy for MOH treatment. The objective of this review is to give a comprehensive synopsis of the literature for the management of MOH. Treatment strategies, such as detoxification and prevention, are the debatable issues. Medication withdrawal is the foundation for management. The available literature suggested abrupt withdrawal with preventive approaches for early management. Bridging therapy could be useful to get relief from withdrawal symptoms. Multidisciplinary choices proved beneficial in supporting withdrawal and preventing relapse. Worldwide, the termination of overused medications has been observed as a standard treatment strategy; however, patient-specific approaches should be taken.

## Introduction

Medication-overuse headache (MOH) is one of the forms of headache mentioned in the Global Burden of Disease (GBD). MOH ranks 18th according to the GBD metric of years of life lived with disability (YLDs) (1). It occurs as a result of frequent and prolonged use of symptomatic medications. MOH usually occurs in chronic migraine patients during the transition from episodic to chronic presentation. MOH may also occur as a result of inadequate treatment of pre-existing headaches (2–4). Despite not being as prevalent as other forms of headaches like tension-type headache (TTH) and migraine, it is a more debilitating and disabling condition. In comparison to other types of headaches, MOH sufferers report negative consequences such as education, career, earnings, social acceptance, and a sense of control over their headaches. Moreover, they do report higher degrees of productivity impairment in housework, professional activities, and social life (5). Hence, the burden of MOH can be considered untoward because it is preventable and treatable.

Increased use of medications could be the patients’ response to amplified pain. However, it can be reasoned that medication overuse cannot be a risk factor for headache aggravation (6). Consumption of pain medications on a daily basis without definitive improvement may reflect ineffective treatment. It results in switching between treatments and discontinuation of prescribed medications (7).

Based on the severity and complexity of MOH, its treatment could be carried out in primary, tertiary, or specialty care settings. In primary care, main attention needs to be given to patient education and advice to minimize medication use (8). In specialty care, detoxification should be the primary objective (9). Furthermore, detoxification protocols differ in two ways: (1) either sudden and complete withdrawal of overused medications or gradual withdrawal; (2) the initiation time for the preventive options (7). It is noteworthy that treatments for MOH are mostly based on expert opinions as compared to scientific evidence. The reason behind the lack of robust scientific evidence for MOH treatment is the exclusion of real-world patients from the clinical trials, and perhaps too much interference by the pharmaceutical industries (10). Therefore, results from randomized clinical trials might not be applicable to patients seeking treatment at tertiary care centers (11). Current treatment failures might be due to a lack of broad and systematic strategies implemented in the past for the treatment of MOH patients. It may corroborate the necessity of more consensual and multidisciplinary approaches. Future trials should consist of applying comprehensive methods, simplified or objective explanations, and using additional accessories to motivate patients to achieve improved outcomes (12).

Following are the diagnostic criteria for MOH according to latest definition of medication-overuse headache (ICHD-3): (1) 10 days per month of ergotamine, triptans, opioids, or combination analgesics on a regular basis for >3 months; (2) simple analgesics or any combination of ergotamine, triptans, and analgesic opioids on ≥10 days per month on a regular basis for >3 months without overuse of any single class alone (13).

In different countries, different strategies are being followed for the treatment of MOH. According to European guidelines, patients should be educated first, followed by preventive medication and withdrawal (11). According to Danish guidelines, medication should be stopped for 2 months. Also, it was recommended to implement pharmacological therapy, if required, after the withdrawal duration (14). Studies have reported that success and adherence to MOH treatment depend on the types of overused medications, the prevalence of psychiatric comorbidities, and bridging the withdrawal phase (7, 10). Real-world comparisons could be difficult among different geographical regions due to cost, variable effect size, and cultural differences.

## Abrupt withdrawal or gradual tapering?

Most of the research found that the choice for abrupt withdrawal was an advantageous strategy for non-opioid analgesics, triptans, caffeine, and ergotamines as compared to barbiturates or opioids due to the risk of the development of withdrawal symptoms (15–17). A study conducted by Hering and Steiner (18) was the first to report beneficial effects after abrupt withdrawal of analgesic drugs and ergotamines. Abrupt and complete withdrawal of analgesics and ergotamines supported by bridging therapy produced a beneficial long-term outcome for 5 years (19, 20). Randomized trials conducted at the Danish Headache Center showed that abrupt withdrawal was more effective in reducing disability and improving quality of life (QoL) as compared to gradual withdrawal (21, 22). Withdrawal headache for triptans, ergotamines, and analgesics are 4, 7, and 9.5 days, respectively, which indicates that withdrawal headache occurs for a short duration for triptans (23). Symptoms associated with opioid withdrawal could last up to 10 days, with multiple effects including nausea, vomiting, headache, anxiety, restlessness, sleep disturbance, and tachycardia (24). These symptoms usually persist for about 2–10 days; however, these do not persist for more than 4 weeks (25, 26). Attention should be given to improving the knowledge of patients about the scope of improvement. Patients should be educated about the possibility of partial success with the withdrawal treatment, as well as the importance of having realistic expectations (27).

The addition of preventive treatment to the complete withdrawal of medications is a confounding issue. Studies have reported that withdrawal is a paramount choice for the treatment of MOH (21, 28, 29). Complete withdrawal is the crucial point; hence, preventive medications should not be required or justified initially (30). However, observational studies reported that prophylactic or preventive treatment could be added in the initial phase to attain complete withdrawal (31, 32). Preventive medications such as sodium valproate, propranolol, topiramate, amitriptyline, flunarizine, and selective serotonin reuptake inhibitors (SSRIs) should be initiated at the start of early discontinuation, during hospital stay, or at the time of hospital discharge (33–35). Early discontinuation of prophylactic medications among medication overuse patients proved beneficial in significantly improving all the parameters, including headache frequency, analgesics used, and the Migraine Disability Assessment (MIDAS) score after follow-up for 6 months, 1 year, 3 years, and 5 years. Functional improvement was evident after follow-up for 5 years (34, 36, 37).

Medication withdrawal without preventive medication resulted in mixed results for MOH outcomes. Medication withdrawal for 2 months with the provision of rescue medication (levomepromazine) only for the initial 1 week produced improvement in headache frequency among 45%, no change among 48%, and increased headache frequency among 7% of patients (38). In another two studies, the discontinuation rate of medications (76%–79%) and reduction in headache frequency (60%–70%) were high (39, 40). In these studies, medication withdrawal was without preventive agents except rescue medications in the form of antiemetics and simple analgesics. The withdrawal without preventive pharmacological agents produced an improved effect among simple MOH patients as compared to the complicated MOH patients. The medication discontinuation rate was 92% among simple MOH patients in comparison to 65% among complicated MOH sufferers (39). Another comparison was made between preventive medications and medication withdrawal (41). It was evident that the headache index and response rates were improved among the preventive medication group as compared to the medication withdrawal group. Follow-up strategies are required for improved outcomes among MOH patients. After the achievement of successful withdrawal, newer and improved status quo needs to be implemented. After unsuccessful withdrawal, case revision is necessary with the implementation of newer strategies such as inpatient withdrawal, preventive medication, and bridging therapies (41).

There is a lack of robust evidence available for abrupt and complete withdrawal of the medications. Moreover, there is no clarity on the timing of the withdrawal of medications. Neither the postponed nor the immediate withdrawal of the medications did not demonstrate superiority (10, 42, 43). Large variability was evident among different studies for the early discontinuation of overused medications. These protocols include inpatient and outpatient discontinuation, intravenous hydration, promethazine as rescue medication, and administration of benzodiazepines, metoclopramide, or corticosteroids for withdrawal symptoms.

## Bridging therapies for (withdrawal) headache

Bridging therapy provides relief from withdrawal headaches during the initiation of the detoxification process. Several bridging therapies have been reported for both inpatient and outpatient settings. Long-acting nonsteroidal anti-inflammatory drugs (NSAIDs), including naproxen, proved valuable for relieving pain during the withdrawal duration in the outpatient setting (18, 44). Confounding evidence is available for corticosteroids as a bridging therapy. A randomized trial found no difference in withdrawal headache between the prednisolone, methylprednisolone, and acetaminophen groups and the placebo group (45–47). Also, a meta-analysis did not report beneficial evidence for prednisolone as compared to the placebo group in terms of withdrawal headache (48). Retrospective analysis showed that IV-dihydroergotamine (DHE) facilitated respite from the refractory headache and tolerated drug withdrawal (49). IV DHE also provides relief for a longer duration after complete withdrawal (50). In a retrospective study, IV aspirin proved to produce relief in patients with MOH and proved to be safe, easy to administer, and well tolerated (51). In 70% of patients after 6 months, IV lidocaine provides relief from MOH (52). However, it could be argued that all these findings were based on weak evidence due to a lack of control groups, randomization, and blinding. Hence, precautions should be taken while considering these medications as bridging therapies (Table 1).

**Table 1:** Comparison of different strategies for MOH treatment.

Treatment strategy	Outcomes
Abrupt and gradual withdrawal	Abrupt withdrawal of overused medications is more effective for MOH patients in reducing disability and improving QoL as compared to the gradual withdrawal.
Bridging therapies for (withdrawal) headache	Evidence for bridging therapies is low; hence, precautions should be taken while using bridging therapies for MOH.
Preventive treatment without adjunctive withdrawal	Preventive therapy could be helpful in avoiding complete withdrawal.
Initial vs. postponed administration of prophylactic medication	Confounding findings are evident for prophylactic medication because the selection of prophylactic treatment was dependent on the underlying headache, comorbidities, and earlier medications.
Inpatient vs. outpatient treatment	No marked difference was evident in terms of patient outcome for inpatient and outpatient strategies. However, outpatient strategy proved to be more cost-effective.
Reduction in headache frequency after simple advice	Advice to patients is considered the most cost-effective strategy for treatment and management of MOH.
Non-pharmacologic treatment	Success of non-pharmacological treatment is limited.
Multidisciplinary approach	MOH is a multifactorial condition; hence, a multidisciplinary approach could be more beneficial for MOH.

## Preventive treatment without adjunctive withdrawal

A few findings stated that complete withdrawal is not required when preventive therapy is provided. Topiramate was found to be effective in the treatment of migraine. In European and North American populations, it demonstrated positive outcomes in migraine patients with and without MOH (53). A sub-group analysis of MOH patients treated with onabotulinumtoxin A in a randomized controlled trial for chronic migraineurs revealed positive results in terms of reduction of headache days when compared to the placebo group. The Headache Impact Test-6 (HIT-6) score, which measures headache disability, also decreased (54, 55). Biological therapies such as anti-calcitonin gene-related peptide (CGRP) monoclonal antibodies (mAbs) erenumab and fremanezumab proved advantageous in reducing headache days among MOH patients (56–59). Emerging evidence supports the efficacy of the other mAbs, galcanezumab and eptinezumab, even in patients who do not withdraw (60).

## Initial vs. postponed administration of prophylactic medication

A randomized trial found that starting prophylactic treatment immediately after withdrawal resulted in a significant reduction in headache frequency compared to patients who did not start prophylactic treatment for 3 months after withdrawal (43). However, another randomized controlled trial did not demonstrate significant differences in terms of headache frequency after follow-up for the duration of 3 months, 5 months, and 4 years (61). Two studies did not show changes in headache frequency and dropout rates after administration of prophylactic treatment at the start and after a duration of 2 months (42, 62). After follow-up for 12 months, studies reported that the requirement of prophylactic medications is in the range of 80%–94% for patients receiving prophylactic medication at the start, and its requirement is in the range of 55%–69% for patients with delayed administration of prophylactic treatment (42, 63). In most of the studies, the selection of prophylactic treatment was dependent on the underlying headache, comorbidities, and earlier medications.

## Inpatient vs. outpatient treatment

The treatment setting for MOH should be determined by the individual needs of the patients. Earlier, the outpatient setting was considered the most appropriate and effective first-line approach. Patient education, sudden complete withdrawal, naproxen as a rescue medicine, and prophylactic treatment proved beneficial in 65% of ergotamine overuse patients (44). Improved long-term outcomes with minimal relapse risk after medication withdrawal were evident among outpatients as compared to inpatients (64). Inpatient settings are usually reserved for patients with complicated MOH due to opioid or barbiturate overuse, comorbid psychiatric conditions, and failures in their withdrawal strategies (65). Studies reported that inpatient settings produced favorable outcomes in terms of headache frequency and sustained abstinence from drugs, including ergotamine and barbiturates, following complete withdrawal (19, 66). The inpatient strategy has its own advantages, including advanced treatments in the form of intravenous administration and improved control over painkiller drugs. It suggests that objective treatment could be provided to patients in an inpatient setting rather than relying solely on the patient's description. However, some research showed that a day-hospital setting with bridging therapies could be an alternative to the inpatient strategy (33, 67). Headache frequency was reduced between 39% and 74% among inpatient and outpatient treatments, respectively (61, 67, 68). However, one trial demonstrated significant improvement in headache reduction for inpatient treatment as compared to outpatient treatment for patients with complicated MOH (69). Tassorelli et al. (70), in a multicenter study, demonstrated that headache patterns were the same between inpatient detoxification and outpatient detoxification. However, the dropout rate was higher among outpatient detoxifications. Most importantly, this study reported that patients from four centers in Europe and two centers in Latin America produced similar outcomes. Overall, it was evident that there was no difference in terms of treatment outcomes between inpatient vs. outpatient treatment. However, from an economic point of view, the outpatient strategy proved more cost-effective as compared to the inpatient strategy. It has been estimated that indirect costs are higher for inpatients as compared to outpatients (71).

## Reduction in headache frequency after simple advice

A few studies have found that simple advice can reduce medication intake and reduce headache frequency in MOH patients. People should be informed about the possible risks of medication overuse and MOH reversal after withdrawal of medications. It could be problematic to predict which subpopulation of patients with headache leads to MOH; hence, it is advisable to educate all the patients with headaches about the potential risk of MOH. In many countries, medications such as triptans and opioids are available without prescription, and people are not usually advised about the potential risk of these medications for MOH (72). Hagen et al. reported that simple advice could reduce medication usage by 7 days per month merely by providing advice (41). Grande et al. conducted a study on patients with chronic headache and medication overuse who were interviewed and informed about the potential role of medication overuse on headache. After an 18-month follow-up, it was clear that approximately 77% of patients were not overusing medications, and 44% of patients reported that they did not have chronic headaches (40). Kristoffersen et al. studied the impact of structured, simple advice on patients with MOH. After follow-up for 16 months, 37% of patients persisted with MOH, 30% could not sustain withdrawal of medications, and relapse to MOH was evident in 6.5% of patients (73). Rossi et al. discovered no significant difference in headache frequency between inpatient and outpatient treatment for simple MOH patients following simple advice (62). However, for patients with complicated MOH, no effect of simple advice was evident between inpatient and outpatient treatment, although inpatient treatment among simple MOH patients demonstrated significant improvement in headache as compared to outpatient treatment for simple MOH. In Scandinavian and Northern European countries, simple advice proved effective in augmenting MOH conditions and reducing episodic medication intake. In these countries, socialized medicine is common, with support from paramedical staff and headache nurses. Efficiency in advice and educational programs is more noticeable in these countries due to the minimal use of opioids, barbiturates, and benzodiazepines (74). Patients, as well as healthcare staff, should be educated about the potential risk posed by MOH. It was clear that Norwegian neurology residents had less than expected knowledge of MOH (75). Also, in Sweden, pharmacy professionals demonstrated inadequate knowledge about MOH (76). Hence, it could be argued that these healthcare staff could miss the opportunity to educate patients about MOH. Educational campaigns for general practitioners, pharmacists, specialized doctors, and the public could be beneficial in minimizing the potential risk of MOH. Cross-media educational campaigns in Denmark proved beneficial in improving awareness about MOH (77). In Europe, the European Headache Federation and Lifting the Burden: The Global Campaign Against Headache published a guide for the treatment of headache disorders, with a focus on MOH (78). Advising patients about medication overuse is considered the most cost-effective strategy for treatment and management of MOH (79).

## Non-pharmacologic treatment

A retrospective randomized study by Pijpers et al. reported that patients with support from headache nurses exhibited higher withdrawal as opposed to patients without support (74). Tassorelli et al. conducted a controlled multicenter study and showed that a diary with feedback and an opportunity to contact doctors or nurses helped patients avoid relapse from MOH (80). Headache diaries filled out by patients could also be helpful in maintaining the duration of headache medication and quantity of medication consumed per day. Advanced technology could also be helpful for patients and healthcare providers. Electronic diaries trigger alerts and enable communication for withdrawal and detoxification. Sustained withdrawal could be achieved through motivating patients using video consultation (80, 81). A combined pharmacological treatment with a behavioral approach produced a lessening of MOH relapse. Short psychodynamic psychotherapy subsequent to inpatient withdrawal led to less medication use and reduced relapse. Psychological interventions, including mindfulness, are also advocated for MOH patients. A study carried out among the Italian population demonstrated that headache frequency and use of medication were similar between psychological therapy like mindfulness and prophylactic medication after 3-, 6-, and 12-month follow-ups (82). Patient involvement, aided by behavioral interventions, was critical to the successful management of MOH. Cognitive behavior therapy, stress management, relaxation training, biofeedback, management of comorbidities, enhancement of adherence, and encouragement all contributed effectively to the recovery of MOH patients (83). Acupuncture was compared with topiramate administration in headache patients with or without MOH. From this study, it was evident that the acupuncture group with MOH showed greater efficacy, reduced medication consumption, and disability as compared to the topiramate group (84). However, due to the small sample size, single-center study, and unblinded evaluations, the results of this study could not be considered robust findings. Comorbidities play an important role in exaggerating the MOH condition. Henceforward, effective management of mood disorders, anxiety, concurrent use of psychoactive substances, psychological dependence, and pain catastrophizing could be helpful in accelerating the recovery of MOH patients (85, 86). After 2 months of drug withdrawal, occipital nerve stimulation reduced headache frequency and triptans usage (87). However, another study reported that patients without MOH produced better effects in pain relief as compared to MOH patients after receiving occipital nerve stimulation (88). Occipital nerve stimulation is not recommended because there are less expensive, less invasive, and more efficacious alternatives. It is recommended to evaluate the application of occipital nerve block for patients with MOH because it increases the risk of occipital nerve block (89). Despite these non-pharmacological treatments being well recommended, the probability of success is limited. Consensuses, approaches, and protocols could be helpful for guiding clinicians; however, it could be argued that non-pharmacological therapies could not be implemented among a definite population (7, 43, 80, 82).

## Multidisciplinary approach

Issues associated with MOH patients are multifaceted. Also, related problems such as anxiety and depression triggered by medication withdrawal need to be addressed. Henceforward, a multidisciplinary approach needs to be implemented to sustain abstinence and improve outcomes (90, 91). The advantage of a multidisciplinary approach is based on the combination of different expertise. Occupational workers, physiotherapists, psychiatrists, psychologists, and nursing experts should work together to advocate for several sub-problems. Different aspects such as behavioral, social, and professional should be advocated by the relevant professionals in the field. A multidisciplinary approach proved beneficial to both patients and healthcare providers (92, 93). A real-world patients’ study was conducted in Brazil through a multidisciplinary approach that included providing information about pain, abrupt withdrawal of medications, follow-up with patients, and the initiation of preventive therapy. After 2 and 12 months of follow-up, headache frequency was reduced by 71% and 59%, respectively. The outcome of patients after this simple advice is similar to that of patients from other regions, such as Europe and South America (10). Multidisciplinary treatment programs were also evaluated among the Italian population, comprising different approaches such as only advice to withdraw; advice with prednisolone and preventive treatment; and advice with prednisone, preventive treatment, fluid replacement, and antiemetics. However, there were no significant differences regarding effective withdrawal among different groups (62). In the recent past, the combination of withdrawal and preventive medication proved to be the most beneficial approach for MOH treatment (94). Withdrawal improves response to acute or prophylactic treatment as the headache condition improves (25, 28). A multidisciplinary approach that included detoxification followed by follow-up by physicians, physiotherapists, psychologists, and headache nurses reduced headache frequency and response rate. Most importantly, results from this study conducted at a Danish headache center are noteworthy since the patients recruited were treatment-resistant (77). Combined implementation of discontinuation of overused medications and administration of prophylactic medications proved beneficial in reducing headache frequency and medication use after follow-up for 12 months (42). Bendtsen et al., in a multinational study, demonstrated a significant reduction in MIDAS score and a substantial decrease in the number of patients with depression and anxiety after the implementation of a multidisciplinary approach, including medication discontinuation and prophylactic treatment (95). Multiple assessment factors, including decreased baseline headache duration, medication consumption, improved HIT-6 scores, QoL, depression, and anxiety, were found to benefit from a combination of abrupt medication withdrawal and prophylactic medications (33, 96, 97). In the Medication Overuse Treatment Strategy (MOTS) trial, migraine preventive therapy with or without switching overused medications to alternative medications was compared. This trial demonstrated that migraine preventive medication without switching to alternative medication is not inferior to switching to alternative medication [switching 9.3 (SD 7.2) vs. no switching 9.1 (SD 6.8); *p* = 0.75, 95% CI −1.0 to 1.3] (98).

## Discussion and future direction

MOH is a complex secondary disorder associated with multidimensional factors such as biological, behavioral, and environmental. Its treatment bears many unresolved issues and perhaps potentiates the differences between simple and complex patients (99). The diagnosis of MOH is clinical, based on internationally known criteria. Although emerging literature explores underlying pathophysiologic mechanisms, there is a lack of confirmation tests or biological markers to differentiate between chronic headache and headache due to medication overuse. Available evidence for treatment of MOH is based on expert opinions, retrospective trials, a few prospective analyses, or longitudinal randomized trials. Available studies did not include real-world patients, had a smaller sample size, higher attrition rates, lower statistical power, and higher placebo rates (100, 101).

Inclusive goals for MOH treatment should be long-term benefits with safe, well-tolerated, and efficacious interventions, regardless of geographical areas or countries. Expert opinions, controlled trials, and guidelines are favorable to the withdrawal of overused medications and emphasize the limitations of excessive use of acute therapies (11, 102). The primary goal should be to stop taking medications on a daily basis because they have proven to be effective. However, it could be argued that withdrawal may not be sufficient; it might be necessary to initiate preventive therapy to achieve and maintain favorable outcomes and avoid relapses following withdrawal. Despite the existence of patients, who might not need prevention, the priority is to avoid the return of the overuse pattern (30). Reliable and impartial trials with preventive medications for MOH are still lacking, which results in personal choices for treatments poorly based on scientific evidence. Financial bias is also an issue. The setting for MOH treatments should be based on the high probability of complete and sustained withdrawal. The advancement in the risk-based clinical scoring system to estimate the complex nature of MOH, in addition to the risk of relapse following treatment, could be helpful in differentiating treatment goals among patients. Multidisciplinary approaches with sustained support from healthcare providers can augment withdrawal success (93). A valid answer for an effective MOH treatment could be obtained through dedicated and high-powered clinical trials (28).

## Conclusion

MOH management is multifactorial and varies across regions and cultures. Withdrawal of overused drugs, initiation of preventive treatments with or without bridging, therapies to address headache escalation, and strict follow-up to avoid relapse are crucial aspects. Larger clinical trials involving wider and more realistic patient populations are needed to assure better outcomes. These findings should aid in the understanding of poorly defined, recognized, and undertreated MOH.
